# Operational research within a Global Fund supported tuberculosis project in India: why, how and its contribution towards change in policy and practice

**DOI:** 10.1080/16549716.2018.1445467

**Published:** 2018-03-19

**Authors:** Karuna D Sagili, Srinath Satyanarayana, Sarabjit S Chadha, Nevin C Wilson, Ajay M V Kumar, Patrick K Moonan, John E Oeltmann, Vineet K Chadha, Sharath Burugina Nagaraja, Smita Ghosh, Terrence Q Lo, Tyson Volkmann, Matthew Willis, Kalpita Shringarpure, Ravichandra Chinnappa Reddy, Prahlad Kumar, Sreenivas A Nair, Raghuram Rao, Mohammed Yassin, Perry Mwangala, Rony Zachariah, Jamhoih Tonsing, Anthony D Harries, Sunil Khaparde

**Affiliations:** a Department of Tuberculosis and Communicable Diseases, International Union Against Tuberculosis and Lung Disease, South-East Asia Regional Office, New Delhi, India; b Centre for Operational Research, International Union Against Tuberculosis and Lung Disease, Paris, France; c Independent Senior Public Health Consultant, Nilgiris, Tamil Nadu, India; d Division of Global HIV and Tuberculosis, US Centers for Disease Control, Atlanta, GA, USA; e Epidemiology and Research Division, National Tuberculosis Institute, Bangalore, India; f Department of Community Medicine, ESIC Medical college and PGIMSR, Bangalore, India; g Department of Community Medicine, Government Medical College and SSG Hospital, Vadodara, India; h National Tuberculosis Institute, Bangalore, India; i World Health Organisation India Country Office, New Delhi, India; j Central Tuberculosis Division, Ministry of Health and Family Welfare, Government of India; k The Global Fund to fight AIDS, Tuberculosis and Malaria, Geneva, Switzerland; l Médecins sans Frontières, Brussels Operational Center (LuxoR), Luxembourg City, Luxembourg; m International Union Against Tuberculosis and Lung Disease, South-East Asia Regional Office, New Delhi, India

**Keywords:** operational research (OR), implementation research, Global Fund project, Tuberculosis (TB), India

## Abstract

**Background**: The Global Fund encourages operational research (OR) in all its grants; however very few reports describe this aspect. In India, Project Axshya was supported by a Global Fund grant to improve the reach and visibility of the government Tuberculosis (TB) services among marginalised and vulnerable communities. OR was incorporated to build research capacity of professionals working with the national TB programme and to generate evidence to inform policies and practices.

**Objectives**: To describe how Project Axshya facilitated building OR capacity within the country, helped in addressing several TB control priority research questions, documented project activities and their outcomes, and influenced policy and practice.

**Methods**: From September 2010 to September 2016, three key OR-related activities were implemented. First, practical output-oriented modular training courses were conducted (n = 3) to build research capacity of personnel involved in the TB programme, co-facilitated by The Union, in collaboration with the national TB programme, WHO country office and CDC, Atlanta. Second, two large-scale Knowledge, Attitude and Practice (KAP) surveys were conducted at baseline and mid-project to assess the changes pertaining to TB knowledge, attitudes and practices among the general population, TB patients and health care providers over the project period. Third, studies were conducted to describe the project’s core activities and outcomes.

**Results**: In the training courses, 44 participant teams were supported to develop research protocols on topics of national priority, resulting in 28 peer-reviewed scientific publications. The KAP surveys and description of project activities resulted in 14 peer-reviewed publications. Of the published papers at least 12 have influenced change in policy or practice.

**Conclusions**: OR within a Global Fund supported TB project has resulted in building OR capacity, facilitating research in areas of national priority and influencing policy and practice. We believe this experience will provide guidance for undertaking OR in Global Fund projects.

## Background

The Global Fund to fight AIDS, Tuberculosis (TB) and Malaria (The Global Fund) is an innovative international financing institution forged by governments, civil society, and the private sector to tackle HIV/AIDS, TB and malaria [1]. Every year, Global Fund awards US$4 billion to support public health programmes through project grants aimed to enhance the reach and access of potential life-saving interventions for these three diseases [2]. Global Fund-supported programmes contributed to saving nearly 20 million lives between 2002 and 2015 [3]. The Global Fund is a major international source of funding for TB prevention and care activities in 110 countries. To enhance accountability and efficiency, the Global Fund has earmarked 10% of every project budget for monitoring, evaluation and operational research (OR) [4].

The Global Fund defines OR (also known as implementation research) as – ‘any research producing practical usable knowledge (such as evidence, findings, information) which can improve programme implementation (e.g. effectiveness, efficiency, quality, access, scale-up, and sustainability) regardless of the type of research (design, methodology, approach)’ [5]. There are several examples of OR influencing policy and practice, programme performance and favourable patient outcomes [6].

## Describing ‘why’ and ‘how’ of OR in the Global Fund project in india

In 2009, when this Global Fund project was conceptualized, India was the largest TB burden country in the world with an estimated incidence of 1.8–2.2 million cases of TB, and with approximately 450,000 deaths due to TB [7]. The Government of India was implementing the Revised National Tuberculosis Control Programme (RNTCP) incorporating all elements of the WHO recommended STOP TB strategy. At the national (aggregate) level, the programme’s twin targets of detecting 70% of the estimated new smear positive TB cases (~1.2 million cases) and successfully treating 85% of these cases were being achieved. However, more than half of the districts in the country (375 out of 625 districts) were not achieving one or both of these targets for the past several years [8]. Several operational and implementation challenges were identified as potential causes and research questions pertaining to these challenges were listed as ‘OR priority topics’ by the RNTCP. The RNTCP had established OR committees at the national, zonal (or regional levels) and state level in collaboration with the faculty from various medical colleges to conduct research on these priority topics. However, due to inadequate understanding of the priority topics and processes to undertake OR, sub-optimal capacity, insufficient allocation of funds to undertake and complete OR, and lack of mentorship from experienced researchers, several important OR questions remained unanswered [9].

In 2009, a conglomeration of civil society organisations, led by The Union’s South-East Asia Office (USEA) and World Vision India (WVI), submitted a five-year proposal to the Global Fund Round 9 ‘call for applications’. The proposed project (‘Project Axshya’) aimed to improve the reach and visibility of RNTCP services among identified marginalised and vulnerable communities in India through advocacy communication and social mobilisation (ACSM). The project was approved by Global Fund and initiated in 2010. A full description of the project components is available elsewhere [10].

Project Axshya included three key OR components in addition to various other activities related to ACSM. First, a practical, output-oriented, modular training course to build research capacity of public health professionals. Second, large-scale surveys – at the beginning, middle and at the end of the project period – to assess the changes in TB knowledge, attitudes and practices of the general community, TB patients, health care providers, NGO functionaries and opinion leaders over the project period. Third, OR studies to describe the outcomes/lessons learnt from implementing various Project Axshya ACSM interventions so as to generate evidence to inform policy and practice.

## Implementation of operational research training courses

In collaboration with the Government of India (Central TB Division, New Delhi, and the National TB Institute, Bangalore), World Health Organization India Country Office, U.S. Centers for Disease Control and Prevention (USCDC), The Union co-developed a modular nine-month curriculum featuring coursework on developing appropriate research questions, study design, data collection methods, data analysis, and scientific writing. This training course was similar to the Structured OR training initiative (SORT IT) model developed by the Union and Médecins Sans Frontièrs and then adapted under the leadership of WHO Special Programme for Research and Training in Tropical Diseases [11].

### Selection of participants and research projects

Each OR course began with a call for proposals using a brief one-page concept note outlining the research question, relevance to addressing TB control challenges in India, potential sources of data, and a project timeline (not to exceed 12 months). Twelve to 15 projects were selected in a competitive process for each cohort based upon scientific merit, previous experience, and relevance to the priorities set by the RNTCP. Course participants (approximately 80) included a variety of public health professionals such as in-country RNTCP consultants, State and District TB programme managers, academicians from various Indian medical colleges, and professionals from non-governmental organisations that were supporting the TB control programme in India.

### Training course structure

**Figure 1.**
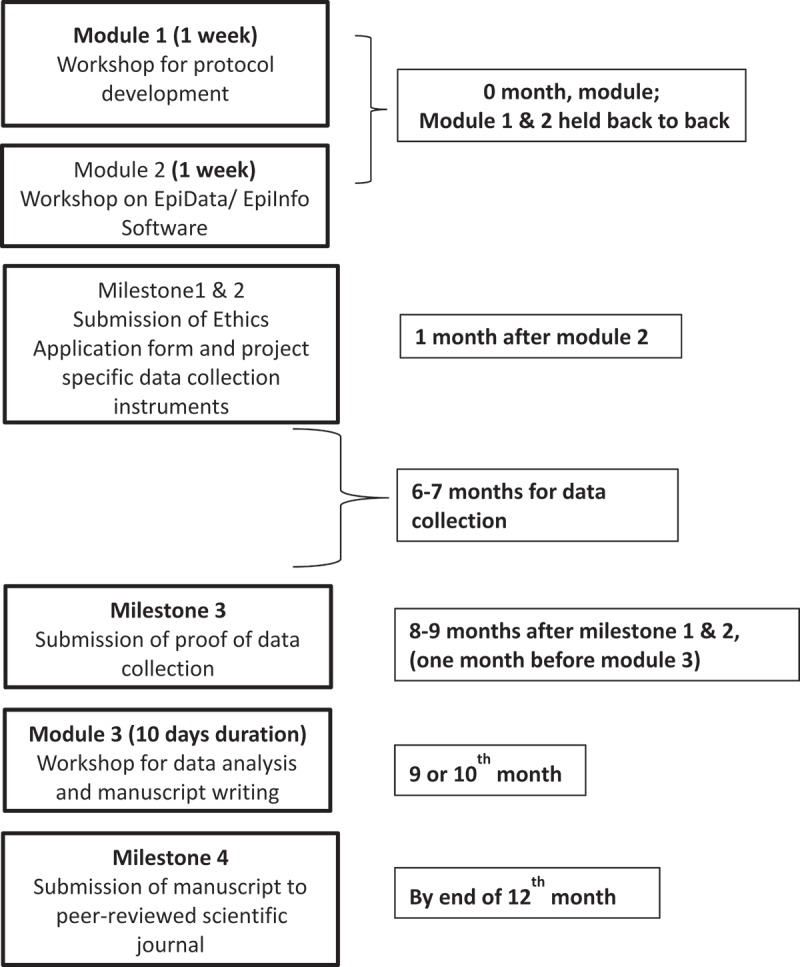
Flow diagram showing the structure of the OR courses undertaken by the Global Fund project in India.

Selected participants, usually working in groups of two to three attended three, one-week modules over a period of nine to 12 months (Figure 1). The modules were a mix of lectures, group discussions and hands-on exercises. The first module focused on developing a research protocol with appropriate study design, and data collection methods that were both scientifically rigorous and ethically sound. At the conclusion of the first module, participants were expected to have a draft protocol, and data collection instruments. The second module, focused on appropriate statistical methods, and use of software packages for quality-assured and efficient data capture and analysis. At the conclusion of this module participants were expected to have created a plan for data collection and data analysis.

Following the first and second module, participants were expected to obtain ethics approval from their respective local ethics committees, ethics committees from the relevant partner organisations such as The Union, National TB Institute (Bangalore) and complete the data collection as per their respective protocols. Only participants who completed data collection were able to advance to the next level.

The third module, typically six-nine months after the second module, focused on data analysis, data interpretation, scientific writing and the peer-review publication process. At the conclusion of this final module, participants were expected to have a draft research paper. Successful completion of the course required a scientific manuscript submitted to a peer-reviewed scientific medical journal no later than 120 days after the conclusion of the third module after obtaining approval from all co-authors.

### Research mentorship

The faculty mentors (six–eight in number) included experienced researchers. Each participant paired with both national (from various Indian institutions) and international (WHO, CDC, The Union) mentors. Most participants had no prior research experience. Active and continual mentorship throughout the course facilitated critical thinking and addressed any problems that arose while implementing the research project. Mentorship was an intensive process of face-to-face consultation, feedback through email exchange, and phone or video conferencing. Faculty took joint responsibility for the teams they mentored and were accountable as co-authors on the peer-reviewed publications.

### Research outputs from the training courses

During April 2010 to Sept 2016, three OR courses were completed and described in this manuscript. The number of applicants selected, the dates of course, and the proportion of studies published in a peer-reviewed scientific journal are shown in Table 1.

**Table 1.** Number of participants, protocols developed, protocols implemented, and peer-reviewed publications by course cohort – Global Fund Project – India, April 2010 – September 2016.

	Training Cohort 1	Training Cohort 2	Training Cohort 3	Total N (%^a^)
Number of applications received	**68**	**42**	**39**	**149**
Number of protocols-teams selected for the training programme	16	14	14	44
Month of first module	Sep, 2010	Mar, 2012	Sep, 2014	
Number of teams in the first module	15	14	14	44 (100%)
Month of second module	Mar, 2011	Mar, 2012	Sep, 2014	
Number of teams in the second module	14	14	14	42 (95%)
Month of third module	Aug, 2011	Jun, 2013	Jul, 2015	
Number of teams in the third module	12	11	11	34 (77%)
Number manuscripts submitted to peer-review*	12	9	9	30 (68%)
Number (%) manuscripts accepted for publication**	12 (75%)	9 (64%)	7 (50%)***	28 (64%)

^a^= denominator is the number of teams selected for the training programme; each team comprised of 2–3 participants in the first two training cohorts and one participant in the third training cohort.

* Includes submission to scientific journals or CDC clearance

** As of 17 September 2017

*** 2 still under review

The proportion of studies published in peer-reviewed journals from the first, second and third courses were 12/16 (75%), 9/14 (57%) and 7/14 (50%), respectively. Overall, when combining all three courses, 44 research protocols were developed, of which 34 (77%) were completed and 28 (64%) were published. Some studies from the second and third courses are still under review, and therefore, the proportion of studies published may increase. The topics investigated varied from course to course. The original research questions and publication status by course are summarized in Tables 2–4.

**Table 2.** OR questions that were undertaken in the first OR course (2010–2011) and the status of their publication in international peer-reviewed scientific journals.

Original OR question/topic	Completed & published
1. What proportion of TB patients would have been additionally diagnosed to have Diabetes Mellitus, if all TB patients are actively screened for DM?	Yes [36]
2. What is the HIV Sero-prevalence among TB suspects (aged 18 years or more) examined for diagnostic smear microscopy at Designated Microscopy Centres (DMCs) of Mandya district, South India?	Yes [30]
3. What is the HIV Sero-prevalence among TB suspects (aged 18 years or more) examined for diagnostic smear microscopy at Designated Microscopy Centres in Vizianagaram district, South India?	Yes [31]
4. Does watching a video of a narrative of cured TB patients (photo-voice) increase adherence to TB medications among new TB patients?	Yes [58]
5. Among pulmonary TB suspects examined for smear microscopy in a DMC, is there an increase in yield of sputum positive cases when the sputum is concentrated by ‘overnight bleach sedimentation’ technique as compared to direct microscopy?	Yes [65]
6. What is the additional yield of TB suspects and sputum smear positive TB cases by intensified case finding among household contacts of TB cases?	Yes [48]
7. Among all smear positive patients registered in 3Q10 what are the factors for delay in initiation of RNTCP treatment after diagnosis in 1 district (Bardhaman) of West Bengal and 1 district (Nalgonda) of Andhra Pradesh?	Yes [51]
8. What is the usefulness of the result of mid CP follow-up sputum smear examinations in declaring outcomes and guiding further management of smear positive TB patients under RNTCP?	Yes [32]
9. Do private practitioners who are exposed to RNTCP involvement efforts report better diagnostic and treatment practices for TB than practitioners who are not exposed with regards to International Standards of TB Care (ISTC)?	Yes [42]
10. Are there any differences in TB management practices by private practitioners in Vishakhapatnam as compared to ISTC	Yes [41]
11. Among TB patients registered under RNTCP what are the patient and provider related factors associated with non-testing for HIV?	Yes [53]
12. What proportion of the diagnosed TB patients in Medical Colleges of West Bengal and Meghalaya, are availing RNTCP treatment services?	Yes [52]
13. What is the duration between onset of symptoms and diagnosis in a cohort of smear positive TB patients diagnosed in the district of Patna by Revised National Tuberculosis Control Programme (RNTCP) and what factors are associated with delay in diagnosis?	No (data not collected)
14. What are KAP among providers of alternate systems of medicine regarding diagnosis, treatment and management of patients with cough as well as chest symptomatic?	No (data not collected)
15. What are the risk factors for death and default among New Smear Positive TB cases in Karnataka?	No (data collected but not analysed and written up)
16. What is the impact of single sputum sample examination during follow ups on management of pulmonary TB patients in RNTCP?	No (data analysed and but manuscript not written up)

**Table 3.** OR questions/topics that were undertaken in the second OR course (2011–2012) the status of their publication in international peer-reviewed scientific journals.

Original OR question/topic	Completed & published
17. Prospective study on inclusion of the family member as a DOT provider for paediatric patients in state of Gujarat	Yes [29]
18. Intensified Case Finding from the Community Level in 10 identified low case detection districts, Odisha, April–September 2012 – a Descriptive Study	Yes [46]
19.Contribution of Mobile Medical Unit for identifying TB suspects and cases in Mohali District, Punjab	Yes [56]
20. Intensified TB case finding at Nutritional Rehabilitation Centres of Bihar, India	Yes [50]
21. Factors for default (loss to follow-up) in drug-resistant TB (DR-TB) treatment: qualitative evaluation of patient and provider reported determinants for DR-TB treatment interruptions in Nagpur, Maharashtra	Yes [47]
22. Isoniazid preventive treatment (IPT) in two districts of Tamil Nadu, India: does practice follow policy?	Yes [49]
23. Introduction of a system of TB case notification among the private practitioners in Pune City: Is it operationally feasible?	Yes [54]
24. Treatment outcomes of MDR-TB patients in Kerala, India	Yes [55]
25. A comparative study on same day sputum smear microscopy with the conventional method in the diagnosis of sputum positive pulmonary TB	Yes [66]
26. Does a real-time web-based patient monitoring system reduce patient drop-outs in the diagnostic and treatment pathway for drug-resistant TB (DR-TB) in Hyderabad district, South India?	No (Published as abstract)*
27. Assessment of the sediment re-decontamination technique in recovering TB bacilli from cultures contaminated on Lowenstein-Jensen medium.	No (paper written but not submitted)
28. Status of MDR-TB suspects after 12–15 months under RNTCP: Programmatic and patient related factors for failure to test MDR-TB	No (Data not collected)
29. Universal access to TB care: Do all TB patients diagnosed in medical colleges come to Revised National TB Control programme?	No (Data not collected)
30. Why do DR-TB patients default in Andhra Pradesh, India?	No (Data not collected)

*Jaju J, Achanta S, Purad CC, Ajay MV Kumar, Ghosh S, Dewan PK, Moonan PK, A Sreenivas. e-SMARTS - Electronic Surveillance and Management of drug Resistant Tuberculosis: An innovative approach towards better patient management in India. Int Tuberc Lung Dis 2013;17(12) Supplement 2:S490.

**Table 4.** OR questions/topics that were undertaken in the third OR course (2014–2015) and the status of their publication in international peer-reviewed scientific journals.

Original OR question/topic	Completed & published
31. Compliance with infection control practices in sputum microscopy centres: a study from Kerala, India	Yes [57]
32. Incidence of Drug Induced hypothyroidism during Intensive Phase of treatment for multi drug resistance TB among patients registered under PMDT in Karnataka, India	Yes [33]
33. Will sensitizing qualified private practitioners and Ayurveda medical officers improve symptomatic referral and TB case detection in Bilaspur District, Himachal Pradesh, India?	Yes [66]
34. TB notification: perspectives and challenges from private health care providers, Delhi – A qualitative study	Yes [67]
35. Has the Policy of implementing decentralized TB diagnostic and HIV testing facilities at PHIs other than DMCs and Integrated Counselling and Testing Centres (ICTCs) – led to increase in TB-HIV case notification in Rajasthan State, India?	Yes [68]
36. Pilot study to explore the feasibility of smoking cessation intervention in smokers with pulmonary TB in RNTCP	Completed, Under peer-review
37. Effect of monitoring DR-TB patients through selected follow-up cultures vis-à-vis the standard schedule among patients treated at Maharashtra, India.	Yes [69]
38. Relationship between nutritional support and TB treatment outcomes among persons living below the poverty-line in West Bengal, India	Yes [70]
39. Knowledge, Attitude and Practices of TB Airborne Infection Control among Antiretroviral Treatment (ART) Centre Health Care Workers	Completed, Under peer-review
40. To assess awareness of pulmonary TB, diagnosis and treatment, as per Standards for TB care in India and perceived constraints in practice of these standards among private practitioners in Cochin city, Kerala, India	Completed (manuscript not written)
41. Is Line Probe Assay (LPA) a valuable tool for testing Extra Pulmonary samples in Programmatic Management of DR-TB: Experience in Kerala State, India	No (Data not collected)
42. Does integration of active follow up of smear negative chest symptomatics not diagnosed as TB, into the routine system through Accredited Social Health Activists (ASHA) increase case detection of pulmonary TB?	No (Data not collected)
43. Will daily Short Message Service (SMS) Reminders Reduce Initial loss to follow up of Smear Positive TB Patients Following Diagnosis? A blinded randomized control trial in one district of South India	No (Data not collected)
44. Association between specific gene mutations conferring Isoniazid mono resistance in pulmonary patients continued on first line of anti-TB drugs and their treatment outcomes in Jharkhand	No (Data not collected)

Common reasons for non-completion included non-feasibility of the study methodology which was realised during data collection, lack of financial support for data collection, limited time to devote to research within current work responsibilities and participants losing interest or moving to other jobs.

## Surveys and other Project Axshya activities related operational research

USEA was directly responsible for conducting three representative KAP surveys at the beginning (2010–2011), mid-point (2013–2014) and at the end of Project Axshya (2017) to assess the changes over the project period in knowledge, attitude and practices pertaining to TB in the general population, TB patients, health care providers, opinion leaders and community-based organisations. The experienced and trained team of technical officers of Project Axshya co-ordinated these surveys. In addition, OR studies aimed to describe some of project’s community-based activities and their outcomes were undertaken and completed by this team of technical officers.

Of the three surveys planned, USEA has completed two till date. The baseline survey was conducted in 2011 in a representative sample of 30 out of the 375 districts. The midline survey was conducted in the same 30 districts in 2013. The detailed reports including methodology of these surveys are available in the public domain [12]. A subset of the data from these surveys was used to answer some specific research questions that were operationally relevant. These studies were written-up as separate manuscripts (n = 6) and published in peer-reviewed journals [13–18]. The findings were also presented at The Union’s World Conferences on Lung Health (years 2011–2016).

OR studies (n = 8) were also done to answer specific research questions using routine monitoring data from the Global Fund Project [19–26]. These studies were besides the studies undertaken in OR training courses and KAP surveys. They pertained to documenting the experiences of active/enhanced case finding in various settings of the country [19–22], describing the various problems identified while servicing the binocular microscopes used by the RNTCP [23], documenting the effects of sensitizing TB patients on their rights and responsibilities as outlined in the ‘patient charter’ [24], using open access technologies to collect data for OR studies using the project’s supervision and monitoring mechanisms [25] and assessing the status of TB control in Indian Prisons [26].

Overall, the three OR activities (three OR courses, two KAP surveys and using routine data from the project) resulted in 42 scientific publications in peer-reviewed scientific journals during the period (April 2010 to September 2017).

## Contribution of OR to policy and practice

**Figure 2.**
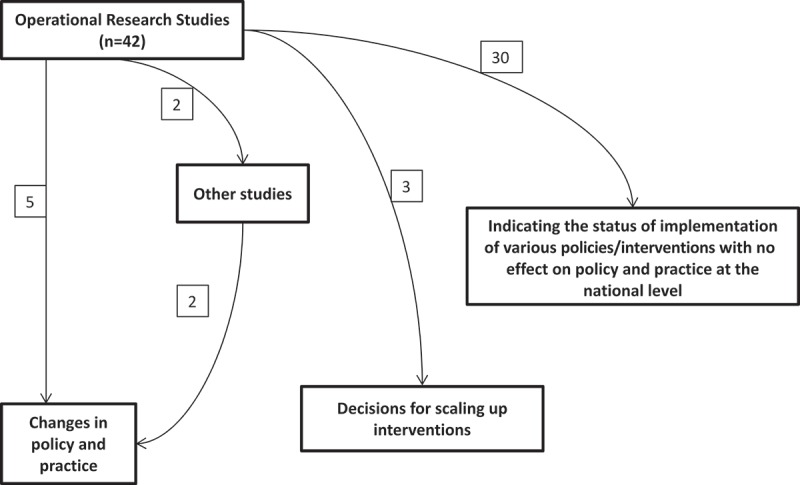
Flow diagram showing the number of OR studies published under the Global Fund project (n = 42) and their pathways for impact on policy/practice. Notes: Five studies had direct impact on changes in policy and practice; Four studies had impact on policies and practices through other studies; Three studies led to decisions for scaling up TB control interventions in the country; 30 studies had limited impact on national level policies.

In order to assess the contribution of these OR studies towards changing policies and practices, we reviewed the two National Strategic Plans (NSP 2012–2017; NSP 2017–2025), and the Revised RNTCP Technical and Operational Guidelines published during the project period [27,28]. We noted instances where our OR was documented as a reference (or included in the narration) as an indication of influencing policy and practice. In addition, we reviewed each project with officials from the RNTCP who are responsible for formulating policies and assessed whether they agreed and endorsed that the study results influenced policy and/or practice. We determined that ~12 studies have contributed to changes in policy and/or practice and describe the pathways towards changes in policy or practice or scale-up of effective interventions in Figure 2.

### Specific examples of OR directly influencing policy

A cluster-randomized, non-inferiority trial conducted in the state of Gujarat showed that the treatment outcomes of pediatric TB patients (TB patients aged <15 years) treated by family members as directly observed treatment (DOT) providers was as good as the outcomes of those treated by the health system designated DOT providers [29]. Upon review, RNTCP considered the evidence to be strong, and revised the national treatment guidelines [27,28]. Throughout India, family members are now eligible to be DOT providers for pediatric TB patients. This change in policy has the potential to reduce the burden of provider-based DOT by ~75,000 patients annually (the number of pediatric TB patients enrolled under the RNTCP each year). Providers can now devote more resources to other TB services.

Studies conducted in Karnataka and Andhra Pradesh showed that HIV testing amongst presumptive TB patients substantially increased the proportion of HIV-positive persons [30,31]. Prior to these studies, HIV counselling and testing of all presumptive TB patients was not a policy in India, despite international recommendations. Following these two studies, the national TB programme recommended universal HIV testing for all presumptive TB patients in high-burden states of the country [27,28]. This policy has the potential to increase HIV counseling testing by 8–9 million people annually (the number of presumptive TB patients assessed for TB each year under the RNTCP).

Laboratory capacity is a major challenge in India. TB diagnosis and patient monitoring requires multiple specimen collection throughout the course of treatment. A study conducted in 43 districts in three large states (Bihar, Karnataka and Maharashtra) showed that discontinuation of mid-continuation phase sputum specimen examinations using smear microscopy did not substantially effect the ability to monitor outcomes [32]. Upon review of these data, the RNTCP revised the national treatment guidelines. As a result, laboratory burden reduced by ~2 million sputum smear examinations (the number of sputa that TB patients enrolled under RNTCP would have otherwise undergone if mid-continuation phase sputum examination was in place). The freed laboratory resources could be transitioned to enhance culture capacity and access to drug susceptibility testing.

Undiagnosed hypothyroidism has serious potential sequelae to Multi Drug-Resistant TB (MDR-TB) patients and increases the risk for additional physical and mental health problems. In India, serologic testing for thyroid-stimulating hormone (TSH), a biomarker for thyroid functioning, is conducted only amongst MDR-TB patients who passively report experiencing signs and symptoms of hypothyroidism during MDR anti-TB treatment. However, hypothyroidism often presents with sub-clinical manifestations that are often masked by other conditions (e.g. arthralgia, depression, ectodermal dysplasia, psychosis, and xeroderma), and hence may be under-diagnosed. A study in Bangalore reported that prevalence of hypothyroidism during MDR treatment was substantially greater when patients had routine serologic testing as compared to passive detection (23% vs 4%) [33]. Data from this study influenced an update of the guidelines to test every MDR-TB patient for hypothyroidism.

### OR studies stimulating further research and collectively contributing to policy change

Diabetes is on the rise in India [34] and has important implication for TB patients [35]. A study in Kerala State in South India identified high levels of diabetes (~45%) among newly diagnosed TB patients [36]. Nearly half of these patients were not aware of their diabetic status at the time of screening. Results from this study and elsewhere contributed to the growing global evidence that diabetes mellitus was a significant risk factor for TB disease and poor TB treatment outcomes. During that time, WHO in parallel, came up with a recommendation to screen all TB patients for diabetes and all diabetes patients for TB [37]. This bidirectional screening was particularly important in India, as it had the highest burden of TB and the second highest burden of diabetes globally [38]. This persuaded the Government of India to endorse nationwide research to assess the feasibility of bi-directional screening at various levels of the health care system [39,40]. Based on these feasibility studies, the RNTCP adopted a policy to routinely screen all TB patients (~1.5 million patients annually) for diabetes mellitus and link them to appropriate care. The benefits of this policy change (in terms of the proportion of TB patients screened and linked to diabetes care, the quality of diabetes care received by these patients, and whether there are any improvements in the TB treatment outcomes) has not been fully evaluated and should be considered in future research.

A publication from the baseline knowledge, attitude and practice survey showed that nearly half of the TB patients in the community were being treated in the private sector, outside the RNTCP notification system [13]. The quality of TB care in the vast un-regulated private sector was largely unknown. Several studies at the same time started highlighting increasing levels of drug-resistant TB from various parts of the country, resulting mainly from inappropriate diagnosis and anti-TB treatment. OR studies undertaken during the same time period in two different cities of the country indicated deficiencies in the private health care providers’ knowledge and practices pertaining to TB diagnosis and treatment [41,42]. At the global level, WHO stepped-up the process of tracking the ‘missing TB cases’ (cases that are not notified to the national TB programmes) and requested all high TB burden countries to enhance their disease surveillance process [43]. Considering all the newly available information and global recommendations, the RNTCP decided to scale up the private sector engagement efforts in the country and also made notification of TB cases diagnosed and treated by the private healthcare providers mandatory [44].

### OR studies contributing to the scale-up of interventions

In 2012, the RNTCP revised its earlier programme targets from detecting 70% of the estimated incident cases and successfully treating 85% of these detected cases to detecting more than 90% of the cases in the community and successfully treating more than 90% of them [45]. In order to achieve this, it identified active case finding among vulnerable and marginalised populations as one of the key strategies. An OR study done as part of the course in the state of Orissa documented that active case finding increased the detection of TB cases by ~10% when compared to control areas [46]. Project Axshya used this study to convince the Global Fund to incorporate active case finding as one of its key activities under the project. The Global Fund agreed and every year ~3.6 million households in marginalised and vulnerable communities in 300 districts are being covered through active case finding. Analysis of this activity for the period April 2013 to December 2014 showed that ~15,000 sputum smear positive cases were detected during active case finding from the marginalised and vulnerable communities [21].

A qualitative OR study done in the state of Maharashtra assessed the patient and provider reasons for MDR-TB patients interrupting treatment. It identified that due to the long course of treatment (~24 months) with drugs that have considerable side effects, these patients may benefit from additional counselling from expert counsellors/motivators [47]. Programme managers of Project Axshya held consultations with various stakeholders and proposed that dedicated counsellors on a pilot basis should provide counselling services for MDR-TB patients in 50 districts of the country. The proposal was accepted by the RNTCP and the Global Fund, and 43 counsellor positions were sanctioned to provide these services in the high MDR prevalent districts. The impact of this intervention in reducing loss to follow-up and mortality rates is under evaluation.

### OR studies indicating the status of implementation of existing RNTCP policies

A large number of studies conducted under this project evaluated the status of implementation of the existing RNTCP policies and guidelines, explored the possible reasons for any deficiencies and provided new insights into the implementation issues. These included: the status of screening household contacts of sputum smear positive pulmonary TB patients [48,49] and malnourished children attending nutritional rehabilitation centers [50], delays in the initiation of TB treatment after diagnosis [51], whether all TB patients diagnosed in the hospitals attached to medical college were being referred and initiated on treatment under RNTCP [52], reasons for poor uptake of HIV counselling and testing [53], status of notification of TB cases by private health care providers [54], TB treatment outcomes of MDR-TB patients [55], detection of TB cases by mobile medical units [56], innovatively addressing the challenges of routine servicing of binocular microscopes [23] and compliance to infection control practices at health facilities [57]. One study assessed innovative ways of improving programme processes such as use of photo-voice for enhancing patient adherence to therapy [58].

## Discussion

OR was made part of the Global Fund grant for building in-country research capacity, documenting project interventions, and influencing TB programme policies and practice through the evidence generated. A recent publication reviewed the situation of OR among Global Fund grantees across six countries and concluded that countries had varied capacities for implementing OR and required more guidance for the same [59]. This is the first time that an experience of promoting OR through a Global Fund grant is documented. During the implementation, several key lessons were learned which are described below.

First, our approach to advancing OR evolved over time and was dependent on epidemiologic, financial, political, and programmatic factors of TB control in India. The priorities of the RNTCP initially posted in 2006 were later updated in 2009 and again in 2012. Prior to our courses, the RNTCP had outlined mechanisms for conducting OR and had earmarked funds for undertaking studies. However, the capacity for research was limited to academic institutions and a few national institutes. There was a large pool of highly motivated young in-country professionals [60] who were supporting the RNTCP in various capacities to implement programme activities. Our courses provided opportunities for this pool of professionals to hone their research skills by helping them identify and frame research questions, understand various study designs, develop scientifically sound protocols, capture and analyze data using statistical software, interpret results, and draft manuscripts.

Second, our approach to developing research capacity had several other important benefits. Our coursework and mentorship empowered local professionals on the frontlines of TB control in India. Developing the skills needed to conduct quality research could have had indirect influence toward improved data quality collected as part of routine activities of the RNTCP, encouraged independent thinking, and facilitated problem solving in practice. Studies conducted by our participants specifically addressed important challenges of RNTCP. The research studies were specifically designed to provide the scientific evidence-base to develop locally-driven solutions and changes to policy and practice.

Third, four participants from previous OR courses were encouraged and provided with opportunities to facilitate in subsequent courses that followed, thereby making them more proficient in conducting OR. This contributed to the creation of a resource pool for OR facilitation within the country. Notably, few of our mentees have become champions for practicing our OR approach and now hold influential positions in TB control in India and elsewhere. In addition, each cohort included at least one CDC Epidemic Intelligence Service Officer (EISO) as co-facilitator and mentor [n = 5] to enrich their learning experience (following a model of ‘see one, do one, teach one’).

Fourth, the structure and content of our courses – modular format, prescriptive milestones, dedicated mentorship, and output-oriented – were similar to the WHO-recommended Structured OR Training-Initiative (SORTIT) courses [11]. However, several notable differences occurred. Many of these differences adapted to local conditions and increased flexibility. For instance, in the first two courses, we encouraged protocols from teams of two to three participants. Our intent was to foster teamwork, increase the number of participants engaged in research, and to ensure continuity of each project (in the event that one or more members was unable to attend all sessions). We accommodated local priorities and needs, both in terms of selection and continuation of participants for the course. We aimed to have participant representation from all states in the country and from institutions not previously engaged in TB research. For example, if a research topic was considered a high priority in any local area, we selected the participants irrespective of qualification or previous experience, especially those from understudied populations. Funding for undertaking research studies did not appear as a bottleneck as most of the studies could be conducted without dedicated funds. In some cases, we linked investigators to funding support available by the RNTCP. Financial support was restricted to attending the training sessions and cost of publication in open access journals. We also increased the duration of the third cohort (to longer than one year) to accommodate for ethics and institutional approvals for primary data collection. Modular milestone deadlines were also flexible when conditions were deemed beyond control of the participant (e.g. postponement of ethics committee meetings, delays in institutional approval of manuscripts or co-authors). Because CDC requires an additional independent review process [61], participants with CDC mentors and co-authors had delayed submission to peer-review. Such participants were considered ‘successful’ upon submission to CDC (rather than to a scientific journal) out of fairness to the participants and maintain our course timelines. With these flexibilities, we hoped to maximize completion rates. Unfortunately, our outputs, when measured in terms of timely completion rates, have not been as impressive as SORT IT courses. SORT IT courses have achieved more than 90% publication rate [62] as compared to 64% publication rate of our courses. The cause of this disparity remains unclear and is an area that requires attention. A recent publication from Rwanda describing the experiences from one OR training course with nine participants (five OR studies) shows how adopting the structured OR training programmes to the local context, with continuous mentoring support from protocol development to scientific publication has resulted in adequate capacity building and completion of research projects in across the country [63].

Fifth, the success of OR in this Global Fund project (in terms of the number of studies conducted or its contribution to policy and practice) is mainly due to engagement and participation of multiple stakeholders who were either involved in providing technical support to the RNTCP or were involved in implementing various TB control activities in the country. In addition, there are several technical committees that guide RNTCP in making changes in policies, practices or scale-up of proven interventions. These committees met at least once every year and had direct access to the OR studies, study investigators and the data. This allowed them to judge the strength of the evidence and make suitable recommendations to the RNTCP. This partially explains why the studies had an impact on national level policies.

We acknowledge some limitations in assessing the contribution of the OR studies on policy and practice. We did not assess the influence of our OR had on local policies and practice at the state, district or facility level. Therefore, the contribution towards policy and practice assessed in our study could be an underestimate. Also, we did not systematically assess the impact that training might have had on conducting future research. There are a few frameworks for assessing the impact of research [6,64]. We found it difficult to apply these methods to our work, as these required setting up several processes before and after OR study implementation to document the contribution of research studies. Future Global Fund awardees could consider these frameworks well in advance of conducting any OR.

## Conclusion

This Global Fund-supported project assisted in OR capacity building, facilitated research in areas of national priority and influenced policy and practice. This is a small but significant step as India moves towards achieving the goals of the ambitious End TB Strategy. We believe this experience will provide guidance for undertaking OR in other Global Fund projects in India and other parts of the world.

## Paper context

OR is important to improve public health programme performance. This manuscript describes the process of incorporating OR in a Global Fund supported TB project in India to build research capacity of TB professionals and generate evidence for improving TB programme performance in India. During 2010–2016, 42 scientific papers were published in peer-reviewed journals. Several OR studies have contributed in improving TB control policies and practices in the country.

## References

[1] Schocken C Overview of the global fund to fight AIDS, tuberculosis and malaria. Cent Glob Dev Washington, DC; 2004 Available from: https://www.cgdev.org/page/overview-global-fund-fight-aids-tuberculosis-and-malaria

[2] The Global Fund The global fund 2016 annual financial report. Geneva; 2017 [cited 2018 1 26]. Available from: https://www.theglobalfund.org/media/6388/corporate_2016annualfinancial_report_en.pdf

[3] The Global Fund The global fund overveiw. [Cited 2018 1 26]. Available from: https://www.theglobalfund.org/en/overview/

[4] World Health Organization (WHO) The global fund. guide to operational research in programs supported by the global fund. [cited 2018 1 26]. Available from: http://www.who.int/hiv/pub/operational/or_guide_gf.pdf

[5] Framework for operations and implementation research in health programmes. [cited 2018 1 26]. Available from: http://www.who.int/hiv/pub/operational/or_framework.pdf

[6] Zachariah R, Ford N, Maher D, et al Is operational research delivering the goods? The journey to success in low-income countries. Lancet Infect Dis. 2012;12:415–12. 22326018 10.1016/S1473-3099(11)70309-7

[7] World Health Organization (WHO) Global tuberculosis report. Geneva; 2011 [cited 2018 1 26]. Available from: http://www.who.int/tb/publications/global_report/archive/en/

[8] Central TB Division TB India 2010 RNTCP Status Report [Internet]. New Delhi; 2010 Available from: http://www.tbcindia.nic.in/showfile.php?lid=2922

[9] Central TB Division and WHO India Country Office Report of the joint TB monitoring mission. India; 2015 [cited 2018 1 26]. Available from: http://www.tbonline.info/media/uploads/documents/jmmdraft2015.pdf

[10] The Union Project Axshya. [cited 2018 1 26]. Available from: https://www.theunion.org/what-we-do/technical-assistance/tuberculosis-and-mdr-tb/project-axshya

[11] Ramsay A, Harries AD, Zachariah R, et al The structured operational research and training initiative for public health programmes. Public Heal Action. 2014;4:79–84. 10.5588/pha.14.0011 PMC4539036 26399203

[12] Project Axshya (The Union South Asia Office) Knowledge, attitude, practices survey reports (Baseline and Midline). [cited 2018 1 26]. Available from: http://axshya-theunion.org/kap-study-report/

[13] Satyanarayana S, Nair SA, Chadha SS, et al From where are tuberculosis patients accessing treatment in India? Results from a cross-sectional community based survey of 30 districts. PLoS One. 2011;6:e24160. 21912669 10.1371/journal.pone.0024160 PMC3166304

[14] Satyanarayana S, Nair SA, Chadha SS, et al Health-care seeking among people with cough of 2 weeks or more in India. Is passive TB case finding sufficient? Public Heal Action. 2012;2:157–161. 10.5588/pha.12.0019 PMC4463060 26392977

[15] Satyanarayana S, Sagili K, Chadha SS, et al Use of rapid point-of-care tests by primary health care providers in India: findings from a community-based survey. Public Heal Action. 2014;4:249–251. 10.5588/pha.14.0061 PMC4533517 26400704

[16] Thapa B, Chadha SS, Das A, et al High and equitable tuberculosis awareness coverage in the community-driven Axshya TB control project in India. Public Heal Action. 2015;5:70–73. 10.5588/pha.14.0105 PMC4525368 26400604

[17] Sagili KD, Satyanarayana S, Chadha SS. Is knowledge regarding tuberculosis associated with stigmatising and discriminating attitudes of general population towards tuberculosis patients? Findings from a community based survey in 30 districts of india. Subbian S, editor. PLoS One. 2016;11:e0147274. 26829713 10.1371/journal.pone.0147274 PMC4734597

[18] Thapa B, Prasad BM, Chadha SS, et al Serial survey shows community intervention may contribute to increase in knowledge of Tuberculosis in 30 districts of India. BMC Public Health. 2016;16:1155. 27835999 10.1186/s12889-016-3807-1 PMC5106771

[19] Reddy KK, Ananthakrishnan R, Jacob AG, et al Intensified tuberculosis case finding amongst vulnerable communities in southern India. Public Heal Action. 2015;5:246–248. 10.5588/pha.15.0048 PMC4682616 26767178

[20] Prasad BM, Satyanarayana S, Chadha SS Lessons learnt from active tuberculosis case finding in an urban slum setting of Agra city, India. Indian J Tuberc. 2016;63:199–202. 27865243 10.1016/j.ijtb.2016.08.006

[21] Prasad BM, Satyanarayana S, Chadha SS, et al Experience of active tuberculosis case finding in nearly 5 million households in India. Public Heal Action. 2016;6:15–18. 10.5588/pha.15.0035 PMC4809720 27051605

[22] Mallick G, Shewade HD, Agrawal TK, et al Enhanced tuberculosis case finding through advocacy and sensitisation meetings in prisons of Central India. Public Heal action 2017;7:67–70. 10.5588/pha.16.0109 PMC5526483 28775946

[23] Chadha S, Nagaraja SB, Prasad BM, et al Innovatively addressing the challenge of maintaining binocular microscopes under tuberculosis programme in India - Is this feasible? Indian J Tuberc. 2016;63:48–50. 27235945 10.1016/j.ijtb.2016.02.004

[24] Prasad B, Rao G, Rajpal J, et al What empowered community can do for TB care? Experience from India. SAARC J Tuberc Lung Dis HIV/AIDS. 2016;13:39–42

[25] Samvad A, Group S, Hd S, et al Data collection using open access technology in multicentre operational research involving patient interviews. Public Heal Action. 2017;7:74–77. 10.5588/pha.15.0079 PMC5515255 28744430

[26] Prasad BM, Thapa B, Chadha SS, et al Status of tuberculosis services in Indian prisons. Int J Infect Dis. 2017;56:117–121. 28179148 10.1016/j.ijid.2017.01.035

[27] Central TB Division Technical and operational guidelines for tuberculosis control in India 2016, revised national tuberculosis control programme. [cited 2018 Jan 26]. New Delhi; 2016. Available from: http://tbcindia.gov.in/index1.php?lang=1&level=2&sublinkid=4573&lid=3177

[28] Central TB Division National Strategic Plan for Tuberculosis Elimination 2017-2025, Revised National Tuberculosis Control Programme. New Delhi; 2017 [cited 2018 Jan 2018 Jan]. Available from: http://tbcindia.gov.in/WriteReadData/NSPDraft20.02.20171.pdf

[29] Dave PV, Shah AN, Nimavat PB, et al Direct observation of treatment provided by a family member as compared to non-family member among children with new tuberculosis: a pragmatic, non-inferiority, cluster-randomized trial in Gujarat, India. Diemert DJ, editor. PLoS One. 2016;11:e0148488. 26849442 10.1371/journal.pone.0148488 PMC4743945

[30] Naik B, Am V K, Lal K, et al HIV prevalence among persons suspected of tuberculosis: policy implications for India. J Acquir Immune Defic Syndr. 2012;59:e72–e76. 22193775 10.1097/QAI.0b013e318245c9df

[31] Achanta S, Am V K, Nagaraja SB, et al Feasibility and effectiveness of provider initiated HIV testing and counseling of TB suspects in Vizianagaram district, South India. Pai M, editor. PLoS One. 2012;7:e41378. 22844467 10.1371/journal.pone.0041378 PMC3402476

[32] Gandhi MP, Am V K, Toshniwal MN, et al Sputum smear microscopy at two months into continuation-phase: should it be done in all patients with sputum smear-positive tuberculosis? Pai M, editor. PLoS One. 2012;7:e39296. 22723991 10.1371/journal.pone.0039296 PMC3378531

[33] Munivenkatappa S, Anil S, Naik B, et al Drug-induced hypothyroidism during anti-tuberculosis treatment of multidrug-resistant tuberculosis: notes from the field. J Tuberc Res. 2016;4:105–110. 27595122 10.4236/jtr.2016.43013 PMC5007858

[34] World Health Organization (WHO) Diabetes country profiles 2016, India [Internet]. Geneva; 2016 Available from: http://www.who.int/diabetes/country-profiles/ind_en.pdf?ua=1

[35] Kumar Nathella P, Babu S Influence of diabetes mellitus on immunity to human tuberculosis. Immunology. 2017;152:13–24. 28543817 10.1111/imm.12762 PMC5543489

[36] Balakrishnan S, Vijayan S, Nair S, et al High diabetes prevalence among tuberculosis cases in Kerala, India. Gordon S V., editor. PLoS One. 2012;7:e46502. 23077512 10.1371/journal.pone.0046502 PMC3471898

[37] World Health Organization (WHO) Collaborative framework for care and control of tuberculosis and diabetes [Internet]. Geneva; 2011 Available from: http://www.who.int/tb/publications/tb-diabetes-framework/en/ 26290931

[38] Stevenson CR, Forouhi NG, Roglic G, et al Diabetes and tuberculosis: the impact of the diabetes epidemic on tuberculosis incidence. BMC Public Health. 2007;7:234. 17822539 10.1186/1471-2458-7-234 PMC2001194

[39] India Tuberculosis-Diabetes Study Group Screening of patients with tuberculosis for diabetes mellitus in India. Trop Med Int Heal. 2013;18:636–645. 10.1111/tmi.12084 23458555

[40] India Diabetes Mellitus–tuberculosis Study Group Screening of patients with diabetes mellitus for tuberculosis in India. Trop Med Int Heal. 2013;18:646–654. 10.1111/tmi.12083 23448175

[41] Achanta S, Jaju J, Am V K, et al Tuberculosis management practices by private practitioners in Andhra Pradesh, India. Pai M, Editor. PLoS One. 2013;8:e71119. 23967158 10.1371/journal.pone.0071119 PMC3742777

[42] Bharaswadkar S, Kanchar A, Thakur N, et al Tuberculosis management practices of private practitioners in Pune municipal corporation, India. Dowdy DW, editor. PLoS One. 2014;9:e97993. 24897374 10.1371/journal.pone.0097993 PMC4045673

[43] Herbert N, George A Baroness Masham of Ilton, et al. World TB Day 2014: finding the missing 3 million. Lancet. 2014;383:1016–1018. 24656187 10.1016/S0140-6736(14)60422-0

[44] Central TB Division Guidance for TB notification in India [Internet]. New Delhi; 2012 Available from: http://www.tbcindia.nic.in/showfile.php?lid=3139

[45] Sachdeva KS, Kumar A, Dewan P, et al New vision for Revised National Tuberculosis Control Programme (RNTCP): universal access - “reaching the un-reached”. Indian J Med Res. 2012;135:690–694. 22771603 PMC3401704

[46] Parija D, Patra TK, Am V K, et al Impact of awareness drives and community-based active tuberculosis case finding in Odisha, India. Int J Tuberc Lung Dis. 2014;18:1105–1107. 25189560 10.5588/ijtld.13.0918 PMC4866500

[47] Deshmukh RD, Dhande DJ, Sachdeva KS, et al Patient and provider reported reasons for lost to follow up in MDRTB treatment: A qualitative study from a drug resistant TB centre in India. Subbian S, editor. PLoS One. 2015;10:e0135802. 26301748 10.1371/journal.pone.0135802 PMC4547708

[48] Khaparde K, Jethani P, Dewan PK, et al Evaluation of TB case finding through systematic contact investigation, Chhattisgarh, India. Tuberc Res Treat. 2015;2015:670167. 26236503 10.1155/2015/670167 PMC4506923

[49] Shivaramakrishna HR, Frederick A, Shazia A, et al Isoniazid preventive treatment in children in two districts of South India: does practice follow policy? Int J Tuberc Lung Dis. 2014;18:919–924. 25199005 10.5588/ijtld.14.0072 PMC4589200

[50] Pathak RR, Mishra BK, Moonan PK, et al Can intensified tuberculosis case finding efforts at nutrition rehabilitation centers lead to pediatric case detection in Bihar, India? J Tuberc Res. 2016;4:46–54. 27066518 10.4236/jtr.2016.41006 PMC4826071

[51] Paul D, Busireddy A, Nagaraja SB, et al Factors associated with delays in treatment initiation after tuberculosis diagnosis in two districts of India. Pai M, editor. PLoS One. 2012;7:e39040. 22792161 10.1371/journal.pone.0039040 PMC3392255

[52] Quazi TA, Sarkar S, Borgohain G, et al Are all patients diagnosed with tuberculosis in Indian medical colleges referred to the RNTCP? [Short communication]. Int J Tuberc Lung Dis. 2012;16:1083–1085. 22668522 10.5588/ijtld.11.0699

[53] Bishnu B, Bhaduri S, Am V K, et al What are the reasons for poor uptake of HIV testing among patients with TB in an Eastern India District? Kranzer K, editor. PLoS One. 2013;8:e55229. 23469163 10.1371/journal.pone.0055229 PMC3585810

[54] Yeole RD, Khillare K, Chadha VK, et al Tuberculosis case notification by private practitioners in Pune, India: how well are we doing? Public Heal Action. 2015;5:173–179. 10.5588/pha.15.0031 PMC4576761 26399287

[55] Duraisamy K, Mrithyunjayan S, Ghosh S, et al Does Alcohol consumption during multidrug-resistant tuberculosis treatment affect outcome? A population-based study in Kerala, India. Ann Am Thorac Soc. 2014;11:712–718. 24735096 10.1513/AnnalsATS.201312-447OC PMC4631605

[56] Binepal G, Agarwal P, Kaur N, et al Screening difficult-to-reach populations for tuberculosis using a mobile medical unit, Punjab India. Public Heal Action. 2015;5:241–245. 10.5588/pha.15.0042 PMC4682615 26767177

[57] Aslesh OP, Ubaid NP, Nagaraja SB, et al Compliance with infection control practices in sputum microscopy centres: a study from Kerala, India. Public Heal Action. 2015;5:255–260. 10.5588/pha.15.0053 PMC4682618 26767180

[58] Shelke SC, Adhav PS, Moonan PK, et al Photovoice: a novel approach to improving antituberculosis treatment adherence in pune, India. Tuberc Res Treat. 2014;2014:302601. 25374679 10.1155/2014/302601 PMC4206923

[59] Kiefer S, Knoblauch AM, Steinmann P, et al Operational and implementation research within Global Fund to Fight AIDS, Tuberculosis and Malaria grants: a situation analysis in six countries. Global Health. 2017;13:22. 28340619 10.1186/s12992-017-0245-5 PMC5366106

[60] Singh RM, Castro KG Rapid assessment of the technical support network provided by the world health organisation to the revised national tuberculosis control programme in India. Health finance and governance project. Abt Associates Inc., Bethesda, MD [Internet]; 2016 Available from: http://www.searo.who.int/india/tuberculosis/topic/rapid_assessment_rntcp.pdf?ua=1

[61] Cono J, Jaffe H The CDC clearance process: supporting quality science. Am J Public Health. 2015;105:e1–2. 10.2105/AJPH.2015.302691 PMC4431110 25879144

[62] Zachariah R, Rust S, Berger SD, et al Building global capacity for conducting operational research using the SORT IT model: where and who? Cipresso P, editor. PLoS One. 2016;11:e0160837. 27505253 10.1371/journal.pone.0160837 PMC4978462

[63] Odhiambo J, Amoroso CL, Barebwanuwe P, et al Adapting operational research training to the Rwandan context: the intermediate operational research training programme. Glob Health Action. 2017;10:1386930. 29119872 10.1080/16549716.2017.1386930 PMC5700541

[64] Bissell K, Lee K, Freeman R Analysing policy transfer: perspectives for operational research. Int J Tuberc Lung Dis. 2011;15:1140–1148. 21943837 10.5588/ijtld.11.0170

[65] Vishnu PH, Bhat P, Bansal A, et al Is bleach-sedimented smear microscopy an alternative to direct microscopy under programme conditions in India? Public Heal Action. 2013;I:23–25. 10.5588/pha.12.0100 PMC4463091 26392991

[66] Bhardwaj RR, Oeltmann JE, Ravichandra C, et al Engaging private providers and Ayurvedic practitioners in Bilaspur, India: did it increase TB case detection? Public Heal Action. 2016;6:154–156. 10.5588/pha.16.0079 PMC4913680 27358811

[67] Satpati M, Nagaraja SB, Shewade HD, et al TB notification from private health sector in Delhi, India: challenges encountered by programme personnel and private health care providers. Tuberc Res Treat. 2017;2017:6346892. 28845306 10.1155/2017/6346892 PMC5563408

[68] Sinha SK, Saxena A, Mishra V, et al Integration and decentralisation of TB-HIV services increases HIV testing of TB cases in Rajasthan, India. Public Heal Action. 2017;7:71–73. 10.5588/pha.16.0060 PMC5526478 28775947

[69] Suryawanshi SL, Shewade HD, Nagaraja SB, et al Unfavourable outcomes among patients with MDR-TB on the standard 24-month regimen in Maharashtra, India. Public Heal Action. 2017;7:116–122. 10.5588/pha.17.0013 PMC5493092 28695084

[70] Samuel B, Volkmann T, Cornelius S, et al Relationship between nutritional support and tuberculosis treatment outcomes in West Bengal, India. J Tuberc Res. 2016;4:213–219. 28042591 10.4236/jtr.2016.44023 PMC5201187

